# Ebstein Anomaly With Right Atrial Clot

**DOI:** 10.14740/cr409w

**Published:** 2015-10-25

**Authors:** Prakash Kumar, Gaurav Singhal, Santosh Kumar Sinha, Umeshwar Pandey, Ramesh Thakur, Chandra Mohan Varma

**Affiliations:** a LPS Institute of Cardiology, GSVM Medical College, Kanpur, UP, 208002, India

**Keywords:** Ebstein anomaly, Right atrial clot, Congenitally unguarded tricuspid valve orifice

## Abstract

Ebstein anomaly (EA) is a rare congenital malformation of the tricuspid valve (TV), often associated with other cardiac malformations, especially atrial septal defect/patent foramen ovale (PFO) which is present in 80-90% of patients and predisposes to paradoxical embolization. We describe the case of a 17-year-old female, who presented with worsening exertional dyspnea, fatigue and pedal edema and atrial fibrillation (AF). Transthoracic echocardiography showed EA with severely dilated right atrium (RA), small functional right ventricle (RV), low velocity flow across TV with spontaneous echo contrast and giant clot in RA. Fortunately for the patient, contrast and transesophageal echocardiography revealed an intact interatrial septum with no PFO preventing any paradoxical embolism from large clot in RA, more so in the background of AF. Important differential diagnosis of congenitally unguarded TV orifice was ruled out due to presence of septal and anterior leaflets of TV and associated chordae.

## Introduction

Ebstein anomaly (EA) is a rare congenital tricuspid valve (TV) malformation which causes “atrialization” of the right ventricle (RV). It represents about 0.5% of congenital heart defects and only 5% of cases survive beyond the age of 50. It is characterized by highly variable clinical manifestations and outcome, which depend on the diversity and severity of the underlying anatomic changes. It is usually associated with other congenital defects, particularly atrial septal defect/patent foramen ovale (PFO), which may be present in 80-90% of patients and predisposes to paradoxical embolism [1].

## Case Report

The authors describe the case of a 17-year-old female admitted with complaints of exertional dyspnea, worsening fatigue and pedal edema. On physical examination, she was hemodynamically stable (blood pressure 136/75 mm Hg, heart rate in atrial fibrillation (AF) with ventricular rate 68 bpm (average), eupneic at rest, with raised JVP with prominent wave and variably split first heart sound and S2 with normal inspiratory split). Laboratory tests, including thrombophilia screen, were normal. Lower limb venous Doppler did not reveal any evidence of deep vein thrombosis. She was not on any central venous lines or any indwelling catheter. The electrocardiogram showed AF (average heart rate 81 bpm). The chest X-ray documented cardiomegaly (CT ratio 70%) due to enlargement of the right atrium (RA).

**Figure 1:**
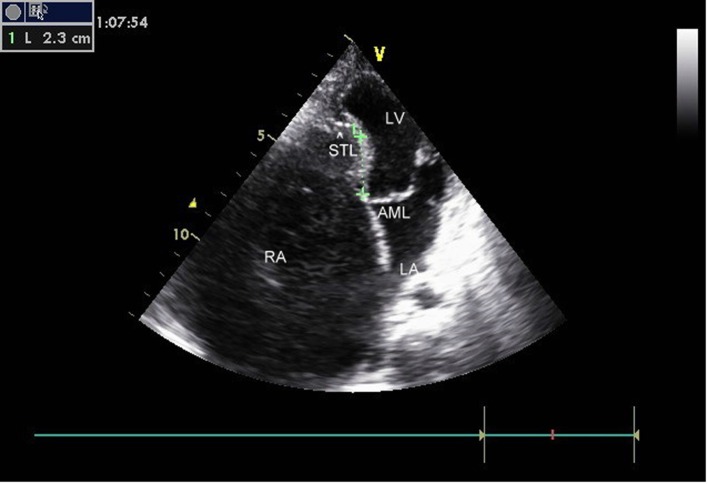
Ebstein anomaly, 2.3 cm apical displacement of septal leaflet (arrow head showing STL, septal leaflet of TV) and massively dilated RA.

**Figure 2:**
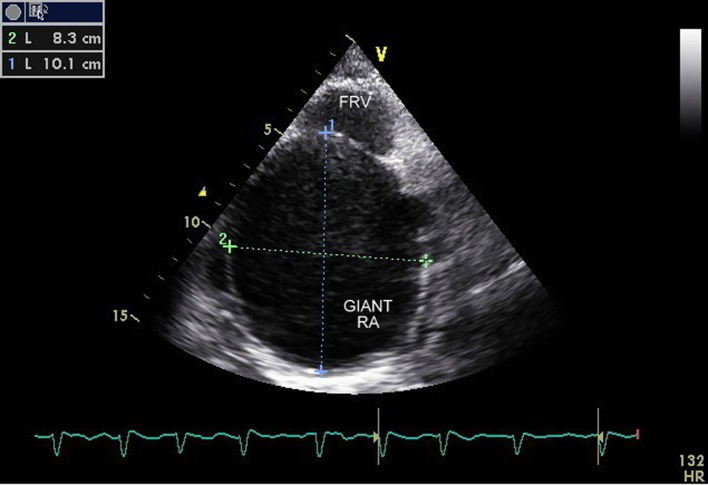
Massively dilated RA (10.1 × 8.3 cm) with small residual functional right ventricle.

**Figure 3:**
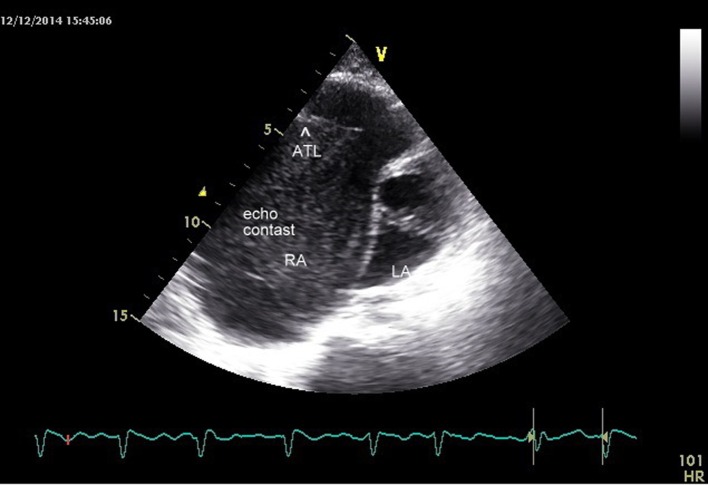
Arrowhead showing elongated ATL (anterior leaflet of TV) with dense spontaneous echo contrast in RA.

**Figure 4:**
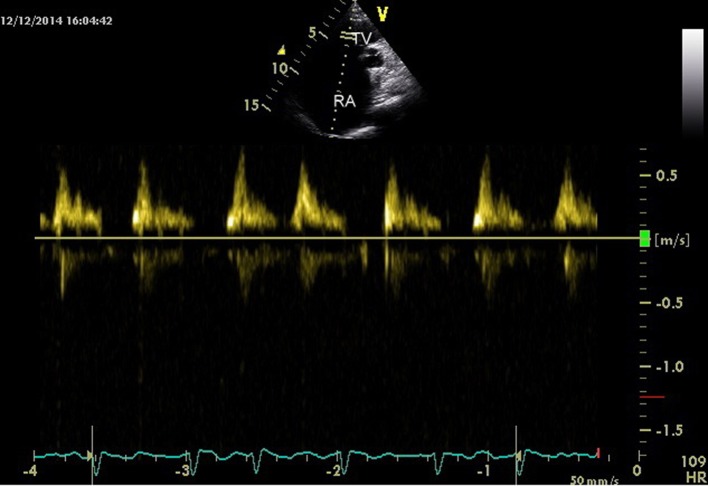
Low velocity flow (0.5 cm/s) across TV.

**Figure 5:**
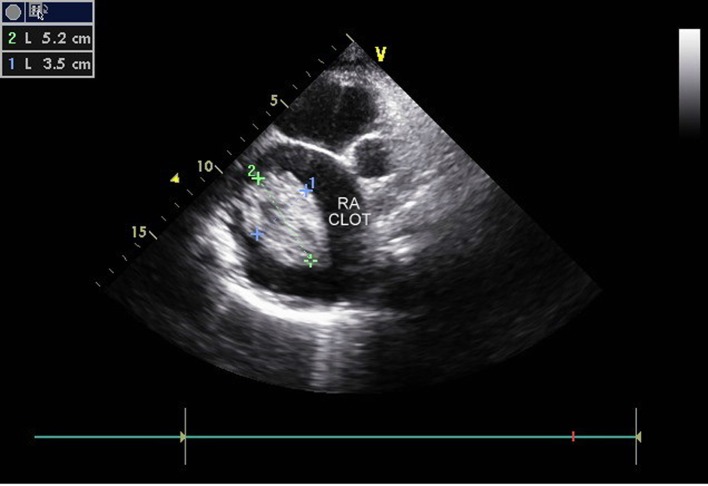
Large clot (5.2 × 3.5 cm) in right atrium.

**Figure 6:**
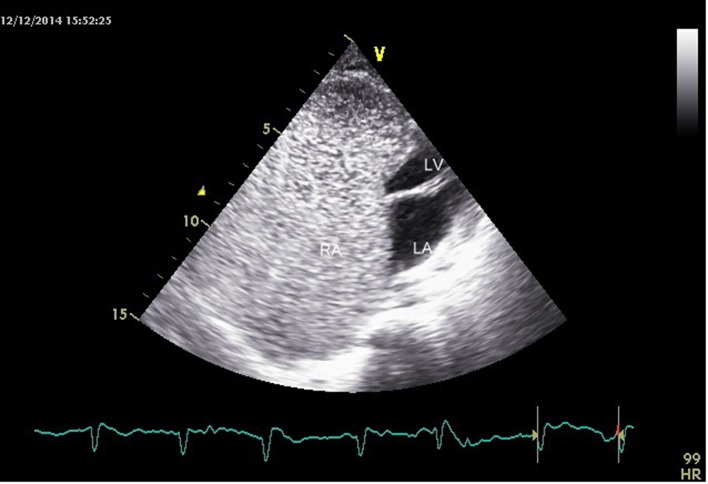
Contrast echo with agitated saline showing no evidence of any PFO with intact interatrial septum (IAS).

**Figure 7:**
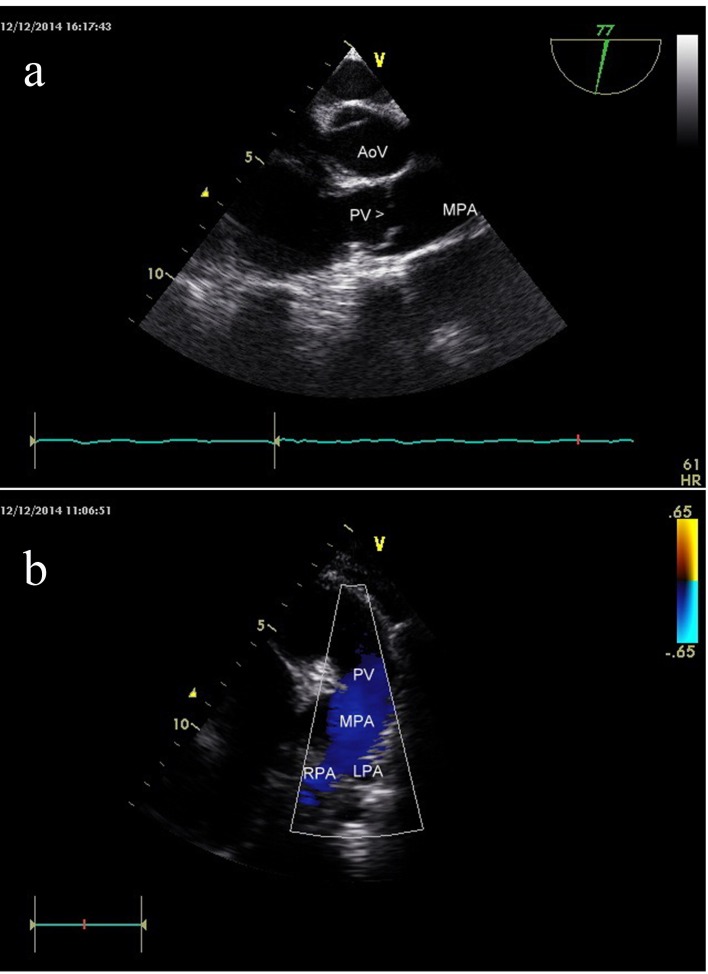
(a) Transesophageal echo showing patent pulmonary valve and pulmonary artery. (b) Transthoracic echo showing patent pulmonary valve and pulmonary artery.

The transthoracic echocardiogram was s/o EA (Carpentier classification type C), showing massively dilated RA, atrialized RV and small and dysfunctional RV (FRV) (Fig. 1, 2, Supplementary Videos 1, 2, www.cardiologyres.org), abnormal TV with 2.3 cm apical displacement of septal leaflet of tricuspid valve (STL) and STL was small, dysplastic and tethered to interventricular septum. Anterior leaflet of tricuspid valve (ATL) was long sail like with decreased movement (Fig. 3, Supplementary Video 3, www.cardiologyres.org), there was stagnation of blood flow in RA due to hypofunctional RV with low velocity flow (1 cm/s) across TV with no evidence of tricuspid regurgitation (Fig. 4), and the massively dilated RA was filled with dense spontaneous echo contrast and a large free floating clot in RA (Fig. 5). Left ventricular systolic function was preserved. Administration of intravenous contrast (agitated saline) revealed no evidence of any PFO with intact interatrial septum (Fig. 6). Transesophageal echocardiography subsequently confirmed the findings with no evidence of a PFO and clearly showed patent normal pulmonary valve and pulmonary artery which was not clearly visualized on transthoracic echo (Fig. 7a, b, Supplementary Video 4, www.cardiologyres.org).

In view of AF and dense spontaneous contrast and large clot in RA, it was decided to start anticoagulation therapy. Patient was advised surgical correction (TV repair, right ventricular plasty and surgical removal of RA clot), but patient defered surgery due to personal reasons.

## Discussion and conclusion

EA in adulthood is often a benign and stable disease, especially in asymptomatic patients [1]. However, clinical manifestations depend on the structural and functional alterations in the right cardiac chambers as well as on TV morphology, and are more severe with greater displacement of leaflet insertion [1]. The clinical presentation can also be affected by other congenital malformations which are often associated, the most frequent of which is atrial septal defect/PFO. In the presence of septal defect, the increased right atrial pressure induced by the hemodynamic and functional changes resulting from EA causes reversed shunting (right-to-left), which is an anatomical substrate for paradoxical embolism that can lead to stroke [1].

Our case had massively dilated RA with dense spontaneous echo contrast with a large clot in RA. Fortunately in our patient, the interatrial septum was intact with no evidence of any PFO even on agitated saline contrast echo, thereby preventing any paradoxical embolism from the large clot in RA, more so in background of AF.

Because of poor RV contractile function, pulmonary circulation is maintained by pumping action of RA or outflow tract which physiologically behaves like functional pulmonary valve atresia resulting from combination of severely abnormal TV and markedly depressed RV contractility. This low velocity passive flow of blood from RA to RVOT (Fontan-like circulation) may have led to stasis of blood and nidus for clot formation in RA.

A close differential diagnosis was unguarded TV orifice which is associated with severe dysplasia of tricuspid leaflets and absence of TV leaflets and absent chordae [2, 3] (presence of septal and anterior leaflets of TV and associated chordae attached to ATL in our case helped in ruling out this). Previous case reports have shown large clot in RA in the setting of unguarded TV orifice [2, 4]. Other differential diagnoses which included pulmonary atresia with intact interatrial septum with TV dysplasia [2] (presence of patent pulmonary valve in our case) and UHL’s anomaly [4] (presence of abnormal TV in our case) were similarly excluded. Right atrial clots are usually seen in association with central venous lines, or indwelling catheters. We report a rare case of EA with a spontaneously formed large clot in RA.
